# Extracellular Vesicles of COVID-19 Patients Reflect Inflammation, Thrombogenicity, and Disease Severity

**DOI:** 10.3390/ijms24065918

**Published:** 2023-03-21

**Authors:** Anat Aharon, Ayelet Dangot, Fadi Kinaani, Mor Zavaro, Lian Bannon, Tali Bar-lev, Anat Keren-Politansky, Irit Avivi, Giris Jacob

**Affiliations:** 1 Hematology Research Laboratory, Hematology Department, Tel-Aviv Sourasky Medical Center, Tel Aviv 69978, Israel; 2 The Sackler Faculty of Medicine, Tel Aviv University, Tel Aviv 6423906, Israel; 3 Department of Medicine F, Tel-Aviv Sourasky Medical Center, Tel Aviv 6423906, Israel; 4 Coagulation Laboratory, Rambam Health Care Campus, Haifa 3109601, Israel; 5 Hematology Department, Tel-Aviv Sourasky Medical Center, Tel Aviv 69978, Israel; 6 Recanati Center, Tel-Aviv Sourasky Medical Center, Tel Aviv 69978, Israel

**Keywords:** COVID-19, extracellular vesicles (EVs), thrombogenicity, inflammation

## Abstract

Severe COVID-19 infections present with cytokine storms, hypercoagulation, and acute respiratory distress syndrome, with extracellular vesicles (EVs) being involved in coagulation and inflammation. This study aimed to determine whether coagulation profiles and EVs reflect COVID-19 disease severity. Thirty-six patients with symptomatic COVID-19 infection with mild/moderate/severe disease (12 in each group) were analyzed. Sixteen healthy individuals served as controls. Coagulation profiles and EV characteristics were tested by nanoparticle tracking analysis (NTA), flow cytometry, and Western blot. While coagulation factors VII, V, VIII, and vWF were comparable, significant differences were found in patients’ D-Dimer/fibrinogen/free protein S levels compared to controls. Severe patients’ EVs displayed higher percentages of small EVs (<150 nm) with increased expression of exosome marker CD63. Severe patients’ EVs displayed high levels of platelet markers (CD41) and coagulation factors (tissue factor activity, endothelial protein C receptor). EVs of patients with moderate/severe disease expressed significantly higher levels of immune cell markers (CD4/CD8/CD14) and contained higher levels of IL-6. We demonstrated that EVs, but not the coagulation profile, may serve as biomarkers for COVID-19 severity. EVs demonstrated elevated levels of immune- and vascular-related markers in patients with moderate/severe disease, and may play a role in disease pathogenesis.

## 1. Introduction 

The emerging novel coronavirus disease (COVID-19), caused by the SARS-CoV-2 virus, is presently the most relevant epidemic health threat. Healthcare centers extensively explored and reported the clinical features of the disease, but its virus pathogenicity remains unclear [1]. SARS-CoV-2 displays a high tropism to epithelial cells, such as pneumocytes, the vascular endothelium, and macrophages. This explains the high incidence of acute respiratory distress syndrome (ARDS)-like features in COVID-19 patients, which is associated with prominent activation of the inflammation–coagulation systems [2,3]. The angiotensin-converting enzyme 2 (ACE2) receptor and transmembrane serine protease 2 (TMPRSS2) play pivotal roles in SARS-CoV-2 infectivity. The coronavirus’ membrane-bound spike (S1) protein binds with high affinity to the membranous ACE2, while the S2 protein is cleaved by the host cell’s TMPRSS2 to allow viral entry into the target cell [4,5]. SARS-CoV-2 entry into host epithelial cells causes the loss of the cellular ACE2 protective (anti-inflammatory, anti-oxidative, anti-apoptotic, and anti-thrombotic) functions, leading to inflammation along with various levels of a cytokine storm, pneumonitis, and endothelial injury [6], resulting in increased procoagulant states in COVID-19 patients. Moreover, increased incidence of thromboembolic events among those with severe disease despite the use of thromboprophylaxis were documented [7]. Therefore, thrombocytopenia and fibrinolysis (high di-dimers, DD) magnitudes are considered significant predictors of mortality [8]. We recently summarized and reported on the role of hyper-fibrinolysis in the inflammation process among patients with COVID-19 [9]. We highlighted the facilitated SARS-CoV-2 cell entry by means of the membranous plasmin (the main product of the fibrinolysis) that has a function similar to that of TMPRSS2, i.e., providing a “plasmin-mediated pathway”.

The increased coagulation–inflammation process in COVID-19 caused by SARS-CoV-2 is related to endothelial and epithelial host cell injuries with the involvement of extracellular vesicles (EVs). EVs include small vesicles (<150 nm, exosomes) and larger vesicles (<1 micron), which shed from the cell surface and express antigens derived from their parental cells [10]. Circulating EVs originating from blood cells and other tissues reflect physiological and pathological states and can serve as biomarkers for diagnosis, treatment monitoring, and disease prognosis [11]. The number of studies showing correlations or associations between EV characteristics and disease prognosis and severity have increased in the last decade. However, in general, the majority of studies on patient EVs are based on a relatively low numbers of subjects [12].

Previous studies have demonstrated that EVs contain cytokines and coagulation factors [13,14] and are involved in hypercoagulation [15,16], inflammation pathways [17], and vascular injury [18] and reflect endothelial damage [19]. We also demonstrated that EVs could reflect disease severity and thrombogenicity in various pathologies, including diabetic vascular complications [19], Alzheimer’s disease [20], and acute myeloid leukemia [21]. EVs serve as novel mediators in the pathogenesis of COVID-19. They facilitate viral spreading via transfer of viral particles and receptors to recipient cells [22] and therefore, should be considered as COVID-19 infectious units [23]. EVs can transfer viral receptors such as ACE2 to recipient cells to facilitate viral infection or directly transport infectious viral particles to target cells, thereby enhancing virus spreading [24]. Several reports have documented an increase in circulating EVs in COVID-19 patients [25], specifically, platelet EVs [26,27]. In addition, there are studies demonstrating the involvement of EVs in the cytokine storm and tissue injury of COVID-19 patients [28]. We therefore wanted to see if EVs can be used as biomarkers for disease severity in COVID-19 patients.

We hypothesized that the magnitude of the inflammatory response of the injured host cells could determine the degree of disease severity in affected individuals and this state may be reflected by the patients’ coagulation profile and EV characteristics. We therefore conducted a study in patients with three different clinical severity levels of COVID-19 to ascertain whether the extent of endothelial cell injury and related inflammatory and coagulation processes can be determined by EVs and their use as biomarkers.

## 2. Results

To define biomarkers that will reflect the inflammatory response magnitude and disease severity in COVID-19 patients, coagulation tests as well as analyses of EV characteristics (EV size, concentration, membrane antigen expression, and cytokine content), were performed.

### 2.1. Patient Characteristics

Thirty-six patients with a COVID-19 infection (confirmed by positive SARS-CoV-2 RT-PCR) were divided into three groups based on disease severity (according to the Israeli Ministry of Health (MOH) criteria): mild (*n* = 12), moderate (*n* = 12), and severe (*n* = 12). The patient characteristics are presented in Table 1. The study also included sixteen healthy controls (HCs). Most of the patients (27, 75%) had a BMI > 25: overweight (*n* = 10 [28%]) or obese (*n* = 17 [47%]). There are no statistically significant differences in terms of age and sex between the three groups. However, the white blood cell count (WBC) was increased in the moderate group compared to the mild group (*p* = 0.049), creatinine levels were higher in the moderate group compared to the severe group (*p* = 0.0142), and the LDH and AST levels were higher in the severe group compared to the mild group (*p* = 0.0230 and *p* = 0.0347, respectively). None of our selected patients had any malignant or premalignant conditions. None of our subjects developed thromboembolic events.

### 2.2. Plasmatic Hemostatic Factors

**Figure 1:**
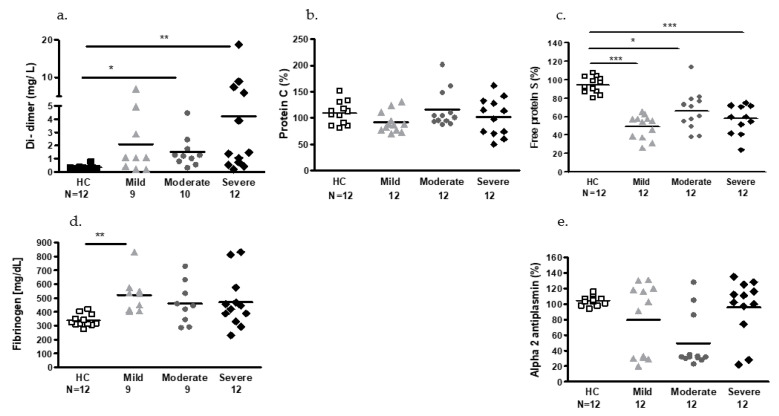
Procoagulant, anticoagulant, and fibrinolytic profiles of COVID-19 patients compared to normal values. (**a**) Normal D-dimer < 0.5 mg/L; (**b**) normal percentage range of protein; C: 70–150%; (**c**) normal range of free protein S: 65–160%; (**d**) normal range of plasma fibrinogen 145–348 mg/dL; (**e**) normal percentage range of alpha2-anti-plasmin (AP) 80–140%. * *p* < 0.05, ** *p* <0.01, *** *p* <0.001.

Procoagulant, anticoagulant, and fibrinolytic profiles of the COVID-19 patients were determined by specific assays, as described in our previous publication [29] and were compared to the normal ranges of each test (Table 1) and to the healthy control (HC) group (Figure 1). The von Willebrand factor (vWF) antigen, factor V (FV), and factor FVIII (FVIII) levels were comparable for all three groups (Table 2). The majority of the COVID-19 patients’ prothrombin time (PT) and partial thromboplastin time (PTT) values were within the normal range (PT 35/36 of the patients; PTT 32/36 of the patients, Table 2). Higher levels of D-dimer were found in the moderate and severe COVID-19 patients compared to the HC group (*p* < 0.05 and *p* < 0.01, respectively). About two-thirds of the COVID-19 patients displayed higher D-dimer levels than the normal range (66% in the mild and 75% in the moderate and severe patients, Figure 1a). The percentage of protein C was found to be similar in the HCs and in the majority of COVID-19 patients (32/36). The percentage of protein C was in the normal range (70–150%). Significantly higher levels of free protein S were found in the HCs (94.06 ± 8.945%) compared to mild COVID-19 patients (46.35 ± 16.85, *p* < 0.001), moderate COVID-19 patients (61.55 ± 26.18%, *p* < 0.01), and severe COVID-19 patients (54.00 ± 20.84, *p* < 0.001). Moreover, about 70% of the COVID-19 patients (25/36) displayed lower values of free protein S, i.e., below the threshold of the normal range (<65%) (Figure 1b,c). Mean fibrinogen levels were similar for all patient subgroups with significantly higher levels in the mild COVID-19 patients (522.7 ± 134.2 mg/dL) compared to the HC group (339.3 ± 43.74 mg/dL, *p* < 0.01). Mean fibrinogen levels were above the normal upper threshold (>348 mg/dL) in most of the COVID-19 patients (100% of the mild group, 88% of the moderate group, and 75% of the severe group) (Figure 1d). No significant changes were found in the levels of Alpha2-anti-plasmin (AP) between the patient groups. However, about 75% of the moderate COVID-19 patients had low AP levels, below the threshold of the normal range (Figure 1e).

### 2.3. EV Characteristics

#### 2.3.1. EV Size and Exosome Markers

**Figure 2:**
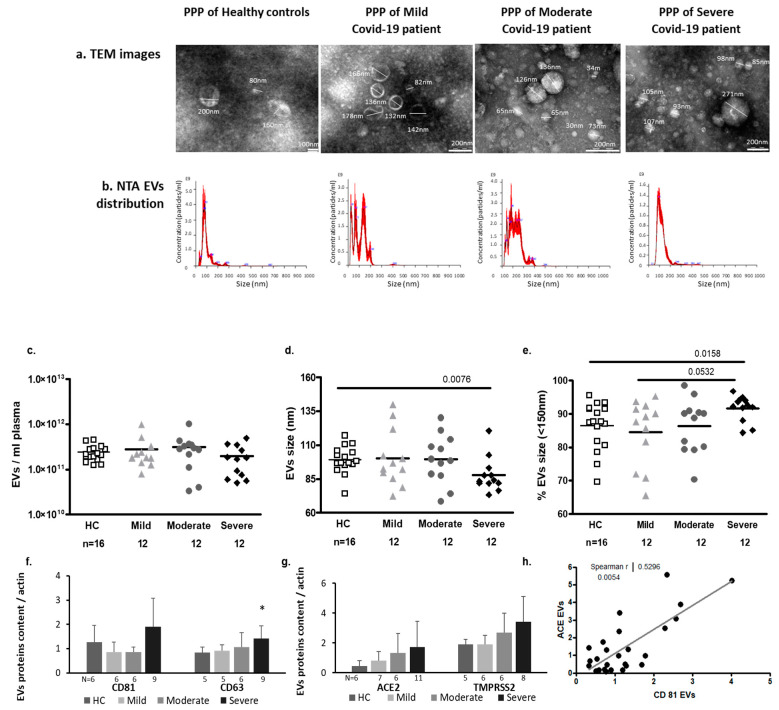
Extracellular vesicle (EV) concentration, size distribution, and expression of exosome markers and markers of viral entry proteins. Platelet-poor plasma (PPP) samples were obtained from the healthy controls and the three patient subgroups: mild, moderate, and severe. (**a**) Transmission electron microscopy (TEM) images of EVs obtained from the study cohorts. (**b**) PPP EV size distribution graph of a representative sample obtained from each of the study cohorts, measured by nanoparticle tracking analysis (NTA). (**c**) PPP EV concentration (particles/mL) and (**d**) mean size distributions were measured by NTA. The graph presents the percentage of small EVs (<150 nm) in each sample (**e**). N = the number of samples that were validated in each subgroup. The expression levels of exosome markers CD63, CD81 (**f**) and SARS-CoV-2 virus entry proteins angiotensin-converting enzyme 2 (ACE-2) and the cell surface transmembrane protease serine 2 (TMPRSS-2) (**g**) were determined by densitometry of a Western blot of samples isolated from the control group and the three COVID-19 subgroups. The graph presents the mean ± standard deviation of each protein expression as a ratio of actin in EV pellets. Gel images are presented in Supplementary Figure SM1. The correlation between the expression of ACE and CD81 is presented in (**h**), * *p* < 0.05.

To ensure that the samples contained vesicles, transmission electron microscope (TEM) images were taken. The images showed EVs in a variety of sizes in all patient subgroups compared to HCs. Nanoparticle tracking analysis (NTA) displayed a similar concentration and size of EVs in platelet-poor plasma (PPP) obtained from the COVID-19 patients and HCs (multivariate analysis, Figure 2a). However, using *t* test analysis, we found that EVs obtained from patients with severe COVID-19 were smaller than the EVs of HCs (87.93 ± 12.76 nm vs. 99.26 ± 10.10 nm, *p* = 0.0076) (Figure 2b). In line with this result, the majority of the EVs obtained from severe COVID-19 patients were smaller than 150 nm (*t* test *p* = 0.0158 Figure 2c) and expressed significantly higher amounts of the exosome marker CD63 (expressed as a ratio of actin) (Figure 2d, Supplementary Figure SM1a–c).

#### 2.3.2. SARS-CoV2 Entrance Proteins ACE2 and TMPRSS2 Expression in EVs

Severe COVID-19 patients’ EVs displayed a trend of increasing levels of ACE and TMPRSS2 compared to HCs, and the size effect analysis displayed large differences between HCs vs. severe COVID-19 patients (ACE: *t*-test, *p* = 0.063, Cohen’s d = 1.025068 and TMPRSS2: *t*-test *p* = 0.0496, Cohen’s d = 0.856734; Figure 2d, Supplementary Figure SM1c,d). Large size effects on ACE expression were also found between the EVs of mild vs. moderate and vs. severe patients (Cohen’s d = 0.873 and Cohen’s d = 0.700, respectively) and between the EVs of mild vs. severe patients in TMPRSS2 EV expression (Cohen’s d = 0.898499). Moreover, ACE expression was found to correlate with exosome CD81 marker expression (r = 0.5296; *p* = 0.0054; Figure 2e).

#### 2.3.3. EV Membrane Antigen Expression

EV membrane antigens were analyzed by flow cytometry using the bead size to set the gate for EV accumulation. An example of membrane antigen expression on EVs obtained from each group is presented in Supplementary Figure SM2.

- Endothelial cell markers

**Figure 3:**
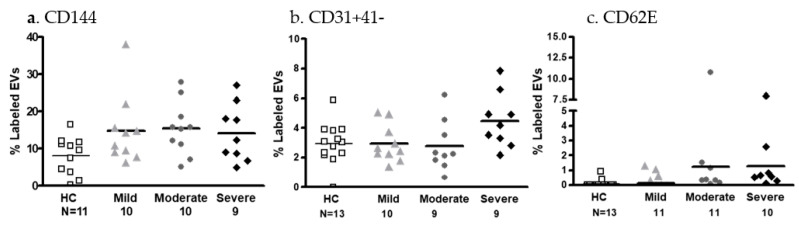
EV endothelial cell markers. The expression of endothelial cell markers on EVs derived from HC PPP and mild, moderate, and severe COVID-19 patients was measured by a CytoFLEX LX flow cytometer. The percentage of labeled EVs was calculated from the total number of EV counts in the vesicles EXo gate, set by the mega-mix beads. The graphs present the percentage of EVs expressing CD144 (VE-Cadherin) (**a**), PECAM-1 (CD31 + CD41-) (**b**), and E-selectin (CD62E) (**c**).

The expression of three endothelial cell markers (CD144, CD31 + 41-, and CD62E) on EVs was found to be similar in the study cohorts (using multivariate analysis). However, t-test analysis showed higher levels of VE-cadherin (CD144) in the moderate COVID-19 patients’ Evs compared to HC Evs (15.38 ± 7.189 vs. 7.928 ± 5.314, *p* = 0.0221) and large size effects (Cohen’s d > 0.9) in CD144 EV expression were seen between HCs and the severe patient subgroups (Figure 3a). In addition, a *t*-test analysis revealed an increase in severe COVID-19 patients’ EVs expressing platelet endothelial cell (EC) adhesion molecules (PECAM-1, CD31 + CD41-; *p* = 0.0452), with a large size effect between patient subgroups (mild vs. severe patients, Cohen’s d = 0.978 and moderate vs. severe patients, Cohen’s d = 0.961).

There was also a trend towards an increase in severe COVID-19 patients’ EVs expressing endothelial–leukocyte adhesion molecule 1 (E-selectin, CD62E) compared to the HCs’ EVs (*p* = 0.0586) with large size effects, when comparing the EVs’ CD62E expression between HCs and moderate and severe patients (Cohen’s d = 0.835) (Figure 3b,c).

- EV platelet markers and coagulation factors

**Figure 4:**
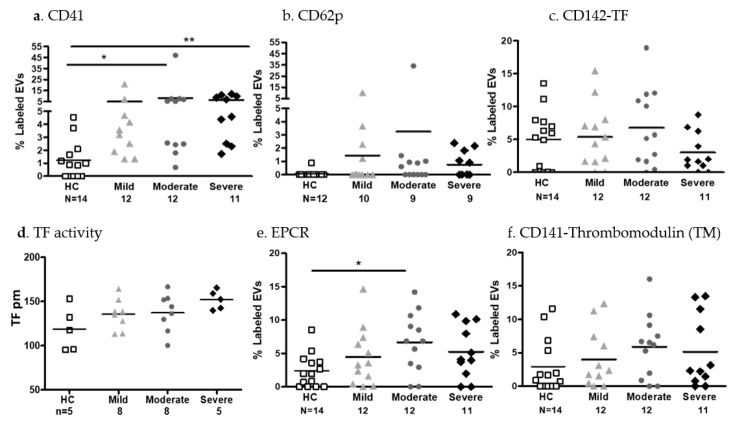
EV platelet markers and coagulation factors. The expression of platelet and activated platelet markers ((**a**) CD41, (**b**) CD62P) on EVs derived from PPP of HCs and mild, moderate, and severe COVID-19 patients, and coagulation antigens, were measured using a CytoFLEX LX flow cytometer. Data are expressed as percentage of labeled EVs in the EXo gate (as described in Figure 2a). Percentages of PPP EVs expressing the procoagulant antigen and tissue factor (TF, CD142) were measured using a CytoFLEX LX flow cytometer (**c**). EV pellet TF activity was measured by a TF Chromogenic Activity Assay Kit (ab108906) (**d**). Percentage of the anticoagulant proteins: endothelial protein receptor (EPCR) and thrombomodulin (TM, CD141) on EVs derived from PPP were measured using a CytoFLEX LX flow cytometer (**e**,**f**) * *p* < 0.05, ** *p* < 0.01.

The EVs of the COVID-19 patients expressed significantly higher levels of platelet antigens (alpha IIb integrin CD41) compared to those of the HCs (*p* < 0.05 for the moderate group, and *p* < 0.01 for the severe group). The expression of activated platelet markers were similar in the multivariate analysis (Figure 4a,b). Levels of EVs expressing the tissue factor (TF) antigen were similar for the three groups with a trend towards a decrease in the severe group’s samples (moderate vs. severe, Cohen’s d 0.793). A TF activity assay revealed that three of the eight samples obtained from severe COVID-19 patients clotted during EV pellet isolation and were therefore excluded from the statistical analysis which showed a significant increase in TF activity in the severe group compared to the HCs (*t*-test, *p* = 0.0556) and to the mild group (*t*-test, *p* = 0.0451) (Figure 4c,d). In addition, the levels of EV expression of EPCR significantly increased in the moderate group (*p* < 0.05) compared to those of the HCs. Thrombomodulin (TM)-expressing EVs were similar in all study cohorts with moderate to large size effects when comparing HC vs. moderate, Cohen’s d = 0.8669, and HC vs. severe, Cohen’s d = 0.60104 (Figure 4e,f).

**Figure 5:**
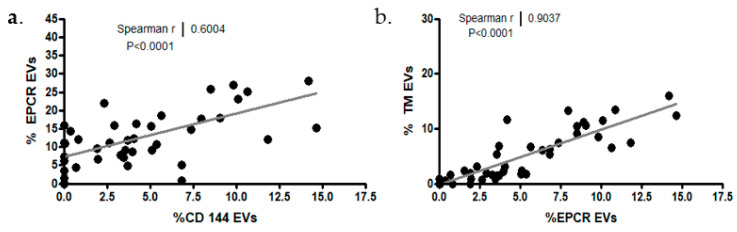
Correlations between expression of EPCR and CD144 (**a**), and between the expression of EPCR and TM (**b**).

High correlations were found in all patients’ EPCR and TM EVs (R = 0.9037; *p* < 0001) and between the percentages of EPCR-expressing EVs and CD144-expressing EVs (R = 0.6004; *p* < 0001) (Figure 5a,b).

#### 2.3.4. EV Immune Cell Markers and Cytokine Content

- EV immune cell markers

**Figure 6:**
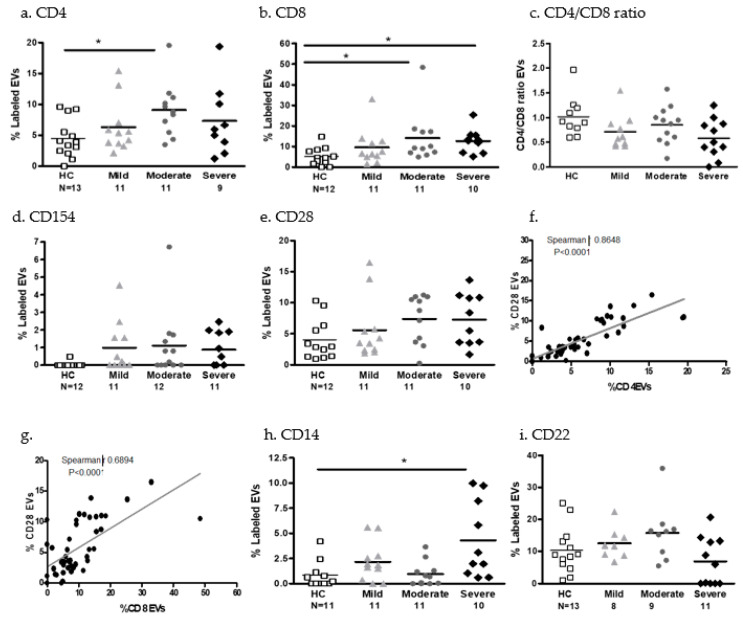
Immune cell marker expression on EVs. The expression of immune cells markers on EVs derived from PPP of HCs and mild, moderate, and severe COVID-19 patients. Data are expressed as percentage of labeled EVs in the EXo gate (as described in Figure 2a) analyzed using a CytoFLEX LX flow cytometer. The graphs show CD4+ helper T cell markers (**a**), CD8+ cytotoxic T cell markers (**b**), the ratio of CD4/CD8 EVs (**c**), CD154 (CD40 ligand) primarily expressed on activated T cells (**d**), and CD28 expressed on T cells which provide co-stimulatory signals for T cell activation and survival (**e**). Correlation between expression of CD28 and CD4 and CD8-labeled EVs (**f**,**g**). CD14, monocyte and macrophage marker (**h**); CD22 B cell marker (**i**). * *p* < 0.05.

The percentage of CD4- and CD8-expressing EVs were higher in the moderate COVID-19 patients (*p* = 0.0077 and *p* = 0.0062, respectively) and CD8-expressing EVs were higher in the severe COVID-19 patients (*p* = 0.0051) compared to the HCs. The overall ratio of CD4+/CD8+ EVs in the mild (*p* = 0.0433) and severe COVID-19 patient groups (*p* = 0.0318) were lower than those in the HC group (Figure 6a–c). EVs expressing T cell activation markers (CD154 and CD28) were higher in moderate and severe COVID-19 patients compared to the HCs (Figure 6d,e). The levels of CD28-expressing EVs highly correlated with CD4- and CD8-expressing EVs (correlation with CD4: r = 0.864, *p* < 0.0001; correlation with CD8: r = 0.6894, *p* < 0.0001) (Figure 6f,g).

Significantly increased levels of membrane antigens were found in the severe COVID-19 patients’ EVs that originated from monocyte or macrophages cells (CD14-expressing EVs, *p* = 0.012) compared to the EVs of the mild COVID-19 patients (*p* = 0.0186) (Figure 6h). The levels of B cell membrane antigens (CD22) were significantly increased in patients with moderate COVID-19 disease compared to the HCs (*p* = 0.0186), but decreased in severe patients compared to HCs (*p* = 0.0276) (Figure 6i).

- EV cytokine cargo

**Figure 7:**
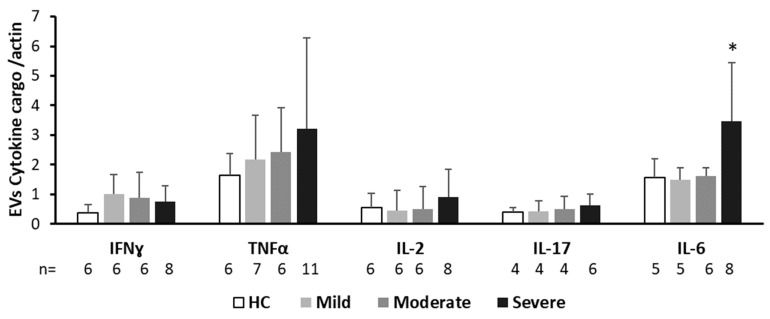
EV cytokine cargo. The expression of cytokines in the EV cargo was determined by Western blot. EV pellets were isolated from the HC group and from the three COVID-19 subgroups. The graph presents the mean ± standard deviation of each protein in the EV pellet: INFϒ, TNFα, IL-6, IL-2, and IL-17. They are expressed as a ratio of actin that served as an unchanged control. Gel images are provided in Supplementary Figure SM1. * *p* < 0.05.

The IL-6 content was twice as high in the severe COVID-19 patients’ EVs compared to the HC EVs (*p* = 0.0451), and also compared to mild and moderate COVID-19 patients (*p* = 0.0186, *p* = 0.0426, respectively) (Figure 6; Supplementary Figure SM1e–g). There was a trend towards an increase in TNFα in the EVs obtained from all three patient subgroups compared to the HCs with moderate-large effect sizes (HC vs. mild COVID 19 patients, Cohen’s d = 0.456; HC vs. moderate COVID 19 patients, Cohen’s d = 0.678; and HC vs. severe COVID-19 patients, Cohen’s d = 0.702). Large size effect differences were found between IFNɣ levels in HC EVs and patient EVs (HC vs. mild COVID 19 patients, Cohen’s d = 1.268; HC vs. moderate COVID-19 patients, Cohen’s d = 0.785; and HC vs. severe COVID 19 patients, Cohen’s d = 0.946). The levels of IL-17 were similar for the patients and the controls, but the size effect analysis displayed large differences between HCs and severe COVID-19 patients (Cohen’s d = 0.73115). In addition, the size effect analysis displayed moderate differences between mild and severe COVID-19 patients in the content of TNF (Cohen’s d = 0.428), IFNɣ (Cohen’s d = 0.424187), IL-2 (Cohen’s d = 0.544), and IL-17 (Cohen’s d = 0.528) (Figure 7 and Supplementary Figure SM1f–h).

## 3. Discussion

The severity of COVID-19 in affected patients is mainly determined by clinical parameters rather than by laboratory tests. It is highly important to have reliable laboratory parameters that will support the decision-making process regarding hospitalization and treatment of COVID-19 patients.

Inflammatory biomarkers can clarify the patient’s condition, which is related to clinical status. For example, protein C (PC) has a prognostic utility and can serve as a biomarker for adult sepsis prognosis. A meta-analysis showed that PC levels are significantly higher in sepsis survivors compared to non-survivors and in patients with sepsis but not with disseminated intravascular coagulation (DIC) [30]. Most of the patients in our study displayed significantly higher D-dimer levels, with the highest level being in the severe group, with lower levels of free protein S and higher fibrinogen levels compared to the HC group (mainly in the mild patient group). However, the levels of all three parameters (D-dimer, free protein S, and fibrinogen) in the patient subgroups were similar, and cannot be used to distinguish between disease severities.

Other laboratory test parameters, including blood cell counts and coagulation profile and chemistry (presented in Table 2), were also similar for the three subgroups. In contrast, we found that EVs could serve as biomarkers for the COVID-19 disease intensity. The EVs of COVID-19 patients with moderate and severe disease revealed changes in endothelial function, coagulation, immune cell response, and inflammation properties. Our study supports recently published studies, albeit based on relatively small groups, showing that the EVs of COVID-19 patients may play a role in endothelial injury, coagulation, and inflammation [31,32,33,34].

In the current study, we found important differences between the EV characteristics of HCs and those from moderate and severe COVID-19 patients. While EV size and concentration were found to be similar in the study cohorts, an increasing trend was found in the percentage of EVs with a size of <150 nm and in exosome markers in severe patients compared to controls that were correlated with ACE expression on EVs. This is in line with previous study results [35,36].

COVID-19 infection results in the loss of ACE function. SARS-CoV-2 enters cells by binding to ACE2 receptors, and activating the renin–angiotensin–aldosterone (RAAS) system. The cleavage of spike proteins by a protease, such as TMPRSS2, facilitates viral entry into the cells. This process leads to shedding of host ACE2 receptors and the loss of its protective function [37].

Loss of ACE2 function leads to upregulation of the RAS/Ang II pathway resulting in vasoconstriction, microthrombosis, endothelial injury, and induction of various inflammatory cascades [6]. An increase in ACE-expressing EVs in COVID-19 patients with severe disease and a trend of increased TMPRSS2 may be indicative of the loss of ACE on the cell surface which leads to endothelial injury and facilitates inflammation. Several studies have shown that EVs which are shed from virus-infected cells contain viral components, including proteins and genetic material [38]. Together with ACE on their surface, COVID-19 patients’ EVs may be considered as viral spreading particles.

### 3.1. EV and Thrombogenicity, Inflammation, and Fibrinolysis

Platelet and endothelial activation were suggested as potential mechanisms resulting in thrombotic complications among COVID-19 patients [39]. In the current study, a non-significant increase was found in endothelial markers, such as platelet PECAM-1 (CD31 + 41-), E-selectin (CD62E), an endothelial cell-specific selectin that is expressed after activation with pro-inflammatory cytokines, and VE-cadherin, which is located on endothelial gap junctions and is required for maintaining the endothelial barrier. An increase in EVs expressing endothelial markers may be indicative of vascular injury that can result in thrombotic complications. However, our study indicates only moderate effects of endothelial EVs.

There is much evidence supporting the association between EV-mediated endothelial apoptosis, endothelial injury, and the inflammation state in patients with COVID-19 [31]. SARS-CoV-2 damages the vascular endothelium, disrupting key roles of the endothelial cells such as anti-inflammatory and anticoagulant functions. When bound to the endothelial protein C receptor (EPCR), the endothelial anticoagulant protein C complex (protein C and S combined and bound to thrombomodulin) is committed to anticoagulant and anti-inflammatory functions. Upon endothelial injury, the soluble form of EPCR (sEPCR) changes its function towards coagulation and inflammation [40]. The translation of the SARS-CoV-2-related endothelial injury into a process of inflammation and intravascular clotting negatively affects the course of the disease. In addition, the presence of very high plasma DD levels is suggestive of hyper-fibrinolysis in patients with severe COVID-19.

We found that platelet EVs were significantly elevated in the moderate and severe COVID-19 patient subgroups compared to HCs as described previously [26], without significant changes in the activated platelet EVs.

EV-TF activity was notably increased in patients with severe COVID-19 compared with mild disease patients and HCs, as previously documented [33]. However, no significant differences were found in the EV-TF expression of COVID-19 patients or HCs. TF is the main activator of the coagulation cascade. It is located in sub-endothelial tissues and is found in the blood circulation in pathological states (e.g., inflammation, sepsis, and cancer). TF is expressed on activated endothelial cells, monocytes, and their EVs and also as a soluble form [41]. TF’s structure, presentation, and expression levels do not always relate to its function [42]. A reduction in TF expression on EV surfaces in the severe group may indicate TF consumption and internalization into the cells. In contrast, EV pellets from PPP probably contained both surface TF and TF that was packaged as cargo inside the EVs, which had a sufficient amount to activate the coagulation cascade. Either way, none of the patients experienced DVT.

To the best of our knowledge, we are the first to describe a significant increase in EVs expressing EPCR in COVID-19 patients with severe disease. EPCR and TM are cofactors that activate protein C (APC), which then cleaves the coagulation cofactors Va and VIIIa, thereby downregulating thrombin generation and serving as an anticoagulant [43]. EVs expressing EPCR may be considered as being part of soluble EPCR (sEPCR) which can bind to APC and reduce its availability. sEPCR is therefore considered a pro-coagulant factor. Moreover, cleavage and release of EPCR from endothelial cells reduces its anti-inflammatory intracellular pathway [43]. In addition, during vascular damage related to infections, sepsis, and inflammation, cytokines from activated leukocytes suppress cell surface expression of TM and EPCR, resulting in reduced levels of APC and an overall increase in thrombogenicity. SARS-COV2 patients display higher levels of sEPCR [44,45,46] and a downregulation of endothelial TM caused by hypoxia that contributes to severe infiltration and coagulopathy in lungs [47]. We assume that the EPCR-expressing EVs are part of the soluble fraction of circulating EPCR.

This study further hints that the measured plasma components of the coagulation system have increased activity in COVID-19 patients. However, the laboratory approach used in our study was not able to show relevant differences in the pro-coagulant and anticoagulant components between the different disease severities. Although the fibrinolytic system showed that its main product, D-dimers, is high in most of the COVID-19 patients but was unrelated to their disease severity. The potent scavenger capabilities of activated plasmin, i.e., alpha2-atiplasmin, was low only in patients with mild-moderate disease severity. This finding indirectly suggests that plasmin is involved in the pathogenicity of SARS-CoV2 infectivity [48].

### 3.2. Immune Cell EVs and Cytokine Cargo

In the current study, COVID-19 patients with severe disease were characterized by high levels of EVs originating from monocytes, B cells, and activated T cells. Previous studies found that changes in COVID-19 disease severity are accompanied by changes in monocytes, macrophages, and B and T cells [3,49]. We demonstrated that changes in EV characteristics with significant increases in EVs expressing CD4, CD8, and CD14, may reflect changes in their parental immune cells. Moreover, the trend of reduction in the CD4/CD8 ratio that was found in the EVs of COVID-19 patients with severe disease was also demonstrated in studies that described the changes in the peripheral lymphocytes and inflammatory cytokines in COVID-19 patients in general [50].

Cytokines can be secreted as soluble factors or as EV-encapsulated forms [51]. We found that the EVs of COVID-19 patients contained higher levels of IL-6, TNFα, IL-2, and INFϒ compared to HCs. The SARS-CoV-2 components (spike and nucleocapsid proteins) trigger the host’s immune system. These viral antigens are recognized by B cells or by other MHC-presenting cells, resulting in antibody production, increased cytokine secretion, and cytolytic activity in the acute infection phase [52]. Clinical reports show that both the mild and severe forms of COVID-19 disease result in changes in circulating leukocyte subsets and cytokine secretion, specifically IL-6, IL-1𝛽, IL-10, TNF, GM-CSF, IP-1, IL-17, and MCP-3 [53]. In the current study, the most significant change in the EVs’ cytokine cargo was related to the IL-6 content in the EVs of COVID-19 patients with severe disease. Monocyte-derived macrophages, which are the first responders to viral infections among the immunoregulatory cells, mainly secrete IL-6 and are the main generators of the inflammatory response in COVID-19 patients [22,54]. We had earlier demonstrated that monocyte-derived microparticles and exosomes induce procoagulant and apoptotic effects on endothelial cells [55]. IL-6 and TNF are linked with fever and with constitutional symptoms, and increase in capillary permeability, hypotension, and acute respiratory failure [53]. We found that increases in IL-6 were early indicators for the progression of mild to severe COVID-19 disease.

The activation of T cells and their ability to produce large amounts of effector cytokines (IL-2, IFNγ, and TNF) was also reflected by EVs obtained from COVID-19 patients with severe disease in the current study. During a SARS-CoV viral infection, T cells recognize the viral antigens presented by MHC class I, which induce cytotoxic activity of CD8+ T cells and MHC class II that present peptides to CD4+ T cells [52]. We also found a trend of increasing EVs expressing CD154+ (CD40L), which is primarily expressed on activated T cells, and in the costimulatory molecule CD28 that were correlated with the increase in CD4- and CD8-expressing EVs in patients with severe disease. These findings support the view that the cell immunity response is increased during COVID-19 infection and promote the inflammation

This study has some limitations. There were no laboratory test results or BMI definitions for the healthy controls. Such criteria were available only to the hospitalized patients, but not for the HCs. We assume that this has only a minor effect on the study results. The plasma volume that could be collected from each patient was limited, and each sample was used in some but not all the experiments.

As described before, studies on EVs are complicated. Their small size requires special conditions for isolation and characterization, and currently, the majority of studies on patients EVs is based on a relatively low number of subjects [12] as was the case in our study which contained a small cohort of patients. Even though COVID-19 is a global pandemic, studies on COVID-19 patients’ EVs are limited and based on small study cohorts. Krishnamachary et al. [32] compared the inflammatory and cardiovascular disease-related protein cargoes of circulating large and small extracellular vesicles (EVs) from 84 hospitalized patients infected with SARS-CoV-2 from different stages and disease severity. Guervilly et al. quantified the EV-TF activity in a cohort of hospitalized patients with COVID-19 (n = 111) and evaluated its link with inflammation, disease severity, and thrombotic events [33].

Future studies on large cohorts will determine if EVs can be used as biomarkers for disease severity related to COVID-19 infection and possibly to other viral infections.

## 4. Materials and Methods

### 4.1. Patient Acquisition

This prospective study was conducted on COVID-19 patients that were admitted to the Internal Medicine Department of Tel Aviv Sourasky Medical Center in Tel Aviv, Israel, a university-affiliated tertiary hospital, between January–April 2021, during Israel’s third wave of the epidemic, which was dominated by the SARS-CoV-2 alpha and beta variants. The study was approved by the local IRB according to the Helsinki principles (Approval No.TLV-401157). For EV characterization, the study also included sixteen HC, age ≥ 18 years, sex- and age-matched, three weeks after receiving BNT162b2 mRNA COVID-19 vaccines, who served as the control group in the study that was registered on clinicaltrials.gov (#NCT04746092). All patients and controls provided informed consent.

#### 4.1.1. Patient Population

Thirty-six consecutive patients were enrolled upon their admission to our internal medicine department after having been diagnosed in the emergency department (ED) as having symptomatic COVID-19. The diagnosis of COVID-19 was confirmed by positive SARS-CoV-2 RT-PCR findings from throat and nasopharynx swabs. The enrolled patients were categorized into three groups according to their disease severity (defined according to the Israeli MOH criteria). We stopped enrollment for each group after reaching 12 patients in each group. Mild illness was defined by a variety of signs and symptoms, such as loss of smell and taste and flu-like symptoms, without shortness of breath, normal chest *X*-rays, and normal SpO2 in room air. Moderate illness was defined by the additional symptoms of lower respiratory diseases (clinical and chest *X*-ray findings), but with a SpO2 level ≥ 94% in room air. Severe illness was defined by symptoms and findings similar to the moderate cases and a SpO2 level < 94% in room air. Patients were excluded if they were critically ill, or had evidence of a bacterial infection, debilitating and critical illness not related to COVID-19, chronic lung disease with low SpO2 levels requiring chronic oxygen support, immune-suppressed conditions, history of clot disorders, use of anticoagulant medication, were unable to sign a consent form, diagnosed as having a thromboembolism event, or were receiving any anti-COVID-19 drugs. All patients provided a detailed medical history and underwent a physical examination, an electrocardiogram, a chest X-ray, and continuous hemodynamic monitoring, and were monitored by closed circuit television. Part of the general blood tests were performed on the ED samples and the rest were done on samples taken upon arrival to the ward, before any medical intervention.

#### 4.1.2. Blood Tests

All laboratory tests are detailed in Table 2. The coagulation parameters prothrombin time (PT), activated partial PT time (aPTT), factor V and factor VIII activities, von Willebrand factor (vWF) antigen, and fibrinogen were measured as described elsewhere [56]. The anticoagulant protein C and free protein S, and fibrinolytic markers (e.g., D-dimer), as well as activities of plasminogen and α2-antiplasmin of each patient were validated as described by Ali-Saleh et al. [57]. The results were compared to standard normal values.

### 4.2. EV Isolation and Characterization

EVs were isolated as previously described [58], according to MISEV2018 [59]. Specifically, platelet-poor plasma (PPP) was obtained after two sequential centrifugations (15 min 1500× *g*, 24 °C) within one hour of collection and frozen in aliquots at −80 °C [60]. The size, concentration, and membrane antigen expression of the EVs were validated on thawed, diluted PPP samples. PPP EV size and concentration were validated by nanoparticle tracking analysis (NTA; Malvern Panalytical NanoSight NS300, Malvern, UK, as described in Supplementary Methods SM1). EV pellets were isolated from thawed PPP by one hour of centrifugation (Centrifuge MIKRO 220R, rotor 1189-A, Hettich, Tuttlingen Germany 20,000× *g*, 4 °C, braking—0). The EV samples that were washed with PBS and pelleted (1 h, 20,000× *g*, 4 °C) were used for transmission electron microscopy (TEM) imaging. Briefly, samples were adsorbed on carbon-coated grids and stained with 2% aqueous uranyl acetate. The samples were examined using a JEM 1400 plus transmission electron microscope (Jeol, Tokyo, Japan). According to the minimal information for studies of extracellular vesicles (MISEV2018 [60]), using fixed samples for TEM is not a quantitative method, as not all particles in a given volume can be imaged, just those that adhere to the grid surface. In addition, the EV pellet cargo was analyzed by Western blot methodology for expression of SARS-CoV-2 entry proteins (ACE-2 and TMPRSS2) and cytokine content was measured by Western blot (Supplementary Method SM2). EV membrane antigen levels were assessed by flow cytometry (CytoFLEX, Beckman Coulter, Indianapolis, Indiana. USA) using fluorescent antibodies (Supplementary Table SM1). Events were collected over time at a flow rate of 10 µL per minute. The controls and samples were analyzed with the same acquisition settings and reagent conditions. Instrument configuration and settings: Gain: FSC 500; SSC 100; Violet SSC 40; PE 120; APC400; FITC 100, threshold: manual 10000 height. EV pellet coagulation activity was validated by the Tissue Factor Activity Assay Kit (Abcam, ab108906, Cambridge, UK).

### 4.3. Statistics

Statistical analysis was performed using the GraphPad Prism 5 software (GraphPad Software Inc., CA, USA). The results were assessed by multivariate analysis, one-way ANOVA, a non-parametric Kruskal–Wallis test, and a Dunn’s post-test that compared all pairs of groups (* *p* < 0.05; ** *p* < 0.01; *** *p* < 0.001). The non-parametric Mann–Whitney U test and Student’s t test were used when only two groups were compared. A *p* value < 0.05 was considered statistically significant. Spearman correlations were performed, along with coefficient value (rho) and 95% confidence intervals. A Fisher’s exact test was used to determine whether or not there was a significant association between two categorical variables. Effect size analysis was performed using Cohen’s d method to characterize the size of the differences between the groups. Small, moderate, and large effects were defined as 0.20, 0.40, and 0.80, respectively [61,62].

## 5. Conclusions

Here, we demonstrated that while routine coagulation blood testing (d-dimer, free protein S and fibrinogen) could distinguish between COVID-19 patients and HCs, these tests were not able to distinguish between the three levels of clinical COVID-19 patients’ disease severity. However, significant changes in the EVs were found not only between healthy controls and patients but also between patient subgroups. The differences were found in several EV membrane antigens including CD41, EPCR, CD4, CD8, and CD14. These markers together with the EV IL-6 content, can serve as biomarkers for disease severity which may better reflect the disease dynamics. EVs are probably involved in the increase in thrombogenicity, endothelial injury, and platelet and immune cell activation, resulting in elevated inflammation in COVID-19 patients with severe disease. We therefore propose that EVs serve as biomarkers for COVID-19 disease dynamics, which will better reflect disease severity than the commonly used plasma coagulation factor levels. Future studies should be performed to support the potential use of EV characteristics as biomarkers for COVID-19 disease intensity. This will hopefully pave the way to establish a methodology to determine disease severity and refine patient management.

## Figures and Tables

**Table 1:** Clinical characteristics of the study patients.

Epidemiological Characteristics				
Characteristic	Mild	Moderate	Severe	*p* Value
Sex				
Male	6	8	7	
Female	6	4	5	
Age	60.6 ± 18.7	60 ± 17.9	62.4 ± 18.4	NS
Body mass index (BMI)	28.5 ± 6.2	29.43 ± 4.27	29.35 ± 7.14	NS
Smoking	0	0	1	NS
Chronic illnesses (total)	20	23	14	
Diabetes mellitus	2	5	4	NS
Hypertension	5	5	0	Severe vs. mild/moderate *p* = 0.037
Dyslipidemia	3	5	2	NS
Cardiovascular	1	2	2	NS
Congestive heart failure	1	1	1	NS
Valvar disease	0	1	1	NS
Atrial fibrillation	2	1	0	NS
Obstructive sleep apnea	2	0	1	NS
Chronic obstructive pulmonary disease	2	0	0	NS
Chronic renal failure	1	2	0	NS
Hyperthyroidism	1	0	0	NS
Hypothyroidism	0	0	2	NS
Immunosuppression	0	1	0	NS
Medication				
Anti-aggregates	2	2	3	NS
ACE-inhibitor, angiotensin receptor blockers	4	1	3	NS
Beta-Blockers	4	1	4	NS
Calcium channel blocker	1	3	1	NS
Proton pump inhibitor (PPI)	2	5	4	NS

**Table 2:** Patients’ laboratory test results.

	Mean ± Std. Deviation	Mild Disease	Moderate Disease	Severe Disease	*p* = *t*-Test
	HB	12.959 ± 2.357	14.139 ± 1.868	13.83 ± 2.2	NS
	WBC	5.818 ± 1.481	9.067 ± 4.131	7.633 ± 3.952	Mild vs. moderate *p* = 0.0489
	LYMPH No.	1.018 ± 0.525	2.417 ± 3.335	1.992 ± 3.333	NS
	NEU No.	4.345 ± 1.196	5.375 ± 2.411	4.933 ± 3.498	NS
	Neu/lymph ratio	6.4 ± 7.468	5.525 ± 3.393	8.7 ± 7.485	NS
	MON No.	0.4545 ± 0.2018	0.6667 ± 0.403	0.525 ± 0.4003	NS
	ESO No.	0.1 ± 0.3	0.1583 ± 0.337	0.05 ± 0.09045	NS
	PLTs	201.5 ± 85.45	268.9 ± 182.6	214.7 ± 163.5	NS
**Coagulation**	Di-dimer	2.099 ± 2.364	1.52 ± 1.199	4.221 ± 5.447	NS
	INR	1.046 ± 0.1041	1.178 ± 0.4686	1.103 ± 0.1155	NS
	PT	10.93 ± 1.07	12.19 ± 4.232	11.53 ± 1.17	NS
	PTT	31.55 ± 6.089	31.8 ± 5.169	29.31 ± 0.8669	NS
	Fibrinogen	522.7 ± 134.2	461.1 ± 150.7	470.7 ± 186.7	NS
	FV	122.6 ± 32.4	135.6 ± 27.49	119.5 ± 32.08	NS
	FVIII	215.2 ± 95.12	255.0 ± 108.8	229.5 ± 80.4	NS
	vWF (IU/dL)	290.7 ± 81.4	356.9 ± 190.8	393.8 ± 211.8	NS
**Chemistry**	Creatinine (Cr.)	1.109 ± 0.7864	1.126 ± 0.9076	0.7333 ± 0.2497	Moderate vs. severe *p* = 0.0142
	Blood urea nitrogen (BUN)	20.9 ± 13	20.75 ± 9.799	19.25 ± 7.569	NS
	Na	137.1 ± 2.548	136.8 ± 4.351	137.9 ± 4.999	NS
	Cl	102.3 ± 3.823	102.5 ± 4.927	101.9 ± 3.848	NS
	K	4.207 ± 0.3957	4.161 ± 0.5111	4.163 ± 0.3994	NS
	Mg	2.201 ± 0.1527	2.042 ± 0.2227	2.103 ± 0.2243	NS
	Ca	8.56 ± 0.4248	8.875 ± 0.6283	8.775 ± 0.4615	NS
	Phosphate	3.173 ± 0.3526	2.863 ± 0.4719	3.043 ± 0.739	NS
	Creatine phosphokinase (CPK)	143 ± 133	106 ± 84	284.3 ± 456	NS
	Lactate dehydrogenase (LDH)	468.7 ± 120.2	679.9 ± 334	750.8 ± 382.3	Mild vs. severe *p* = 0.0230
	Alanine aminotransferase (ALT)	34.91 ± 36.55	61.58 ± 79.3	35.75 ± 22.72	NS
	Aspartate aminotransferase (AST)	31.4 ± 21.55	50 ± 52.17	44.58 ± 16.28	Mild vs. severe *p* = 0.0347
	Alkaline phosphatase (ALKP)	62.7 ± 30.47	72.5 ± 28.66	78.5 ± 37.09	NS
	Gamma-glutamyl transferase (GGT)	41.64 ± 46.08	81.25 ± 75.24	72.75 ± 65.44	NS
	Bilirubin	0.5882 ± 0.337	0.47 ± 0.1473	0.6542 ± 0.4736	NS
	Albumin	38.6 ± 3.921	37.42 ± 4.889	38.58 ± 5.4	NS
	Troponin	11.95 ± 15.16	21.35 ± 46.1	20.83 ± 25.28	NS
	Brain natriuretic peptide (BNP)	27.4 ± 34.5	62.67 ± 16.02	41 ± 27.48	NS
	Ferritin	624 ± 536	1232 ± 926	632.3 ± 598.7	NS
	C-reactive protein CRP)	48.54 ± 50.88	52.33 ± 67.18	76.67 ± 70.02	NS
	Lactate	1.912 ± 1.368	1.687 ± 0.3717	1.97 ± 0.5804	NS
**Gas**	pH	7.401 ± 0.04625	7.415 ± 0.06004	7.381 ± 0.03879	NS
	pCO2	43.88 ± 7.246	39.22 ± 4.98	44.83 ± 9.581	NS
	Bicarbonate (HCO3)	25.54 ± 3.347	24.06 ± 1.538	26.11 ± 3.591	NS
	pO2	26.31 ± 15.07	35.46 ± 15.16	32.48 ± 15.54	NS

non-significant (NS).

## Data Availability

Data is unavailable due to privacy and ethical restrictions.

## Supplementary Materials

The following supporting information can be downloaded at: https://www.mdpi.com/article/10.3390/ijms24065918/s1.
