# Supplementary material for: Extracellular Vesicles of COVID-19 Patients Reflect Inflammation, Thrombogenicity, and Disease Severity
Source: Int J Mol Sci. 2023 Mar 21;24(6):5918. doi: 10.3390/ijms24065918 (PMC10054500; doi:10.3390/ijms24065918)
Supplement: Supplementary file 1 [file ijms-24-05918-s001.zip › ijms-2217026-supplementary.pdf]

Supplementary Methods

Supplementary method (SM)

SM1. Nano tracking analysis (NTA) was performed using NS300 Automated Nanoparticle Characterization Instrument (Malvern Instruments LTD Worcestershire Malvern ,UK)., Software Version build 3.1.54., Camera Type—sCMOS, Laser Module: NS300, 405 nm). Software settings for analysis were kept constant for all measurements. Capture settings were as follows: camera level = 7, slider shutter = 1232, slider gain = 219, number of frames = 749, temperature = 25°C, viscosity = 0.86 cP, and syringe pump speed = 20. Five 30 s videos were recorded per sample in light scatter. Samples were diluted with phosphate buffered saline (PBS) filtered through a 0.1 um membrane. The filtered PBS served as control for each measurement

SM2. Western blot (WB) analysis: A sample of 30ul of EV pellets obtained from similar PPP volume (250ul) were combined with 2X·lysis buffer (Ray Biotech, Norcross, GA, USA) supplemented with 1% proteinase inhibitor and 1% phosphatase inhibitors (Sigma) containing β-mercaptoethanol (1:20, Biorad) (12). Samples were loaded and separated on 4-20% Mini-PROTEAN TGX Precast Protein Gels (Bio-Rad) and then transferred to Trans-Blot Turbo Mini 0.2 μm Nitrocellulose Transfer Packs (Bio-Rad). The transferred membranes were incubated with specific antibodies (supplementary Table 1), documented by myECL™ Imager and analyzed by My Image Analysis Software (both from Thermo Fisher Scientific, Waltham, MA USA).

Supplementary figures and tables

Supplementary Table SM1 Antibodies and labeling kits

**Antibodies for flow cytometry**	**Color**	**Vender**	**Cat. number**
Mega mix beads 0.5,0.9,3um	FITC	BIOCYTEX	7801
IgG	FITC	BD	345815
IgG	PE	BD	400112
IgG	APC	BD	555751
Anti-CD235a (red blood cells)	PE	Dako	R7078
Anti-CD41a (platelet)	APC	BD	559777
Anti-CD62P (activated platelets)	PE	BD	555524
Anti-CD62E (endothelial cells)	APC	BD	551144
Anti-CD 144 (endothelial cells)	APC	Biolegend	B259463
Anti-CD31 (endothelial cells & platelets)	FITC	BD	555445
Anti-CD201 (endothelial protein C receptor (EPCR), coagulation	APC	BD	563622
Anti-CD142 (tissue factor, coagulation)	PE	BD	550312
Anti-CD141 (thrombomodulin, coagulation)	APC	BD	564123
Anti-HLADR (MHC class II)	APC	BD	559868
Anti-CD11a (mediate leukocyte adhesion)	PE	BD	555384
Anti-CD 14 (monocyte, inflammation)	FITC-FL1	BD	555397
Anti-CD4 (T cells)	APC	BD	555349
Anti-CD8 (T-cell receptor (TCR)	APC	BD	566852
Anti-CD28 (T-cell activation)	APC	BD	559770

Anti-CD22, B lymphocyte-specific adhesion molecule	PE	BD	337899
Antibodies and kits for Western blot	Concentration	Vender	Cat. number
Anti-CD63	1:1000	abcam	ab59479
Anti-CD81	1:1000	abcam	ab79559
Anti-interferon gamma (INFγ)	1:1000	abcam	ab133566
Anti-TNF alpha	1:1000	abcam	ab215188
Anti-IL-2	1:1000	abcam	ab9238
Anti-IL-6	1:500	abcam	ab9324
Anti-IL-17	1:1000	abcam	ab79056
Anti-TGFβ	1:1000	abcam	ab215715
Anti-actin	1:1000	Biomedical	691001MP
Anti-mouse	1:5000	Jackson	115-035-146
Ant-rabbit	1:5000	Jackson	111-035-144
WESTAR NOVA 2.0 chemiluminescent substrate for WB		CYANAGRN	9470XLS0710250

* PPP samples were diluted 1:5 with filtered PBS (by 0.1um), labeled with double or single fluorescent antibodies for 30 min in the dark and ending with the adding of FACS buffer (PBS with 0.5% PFA).

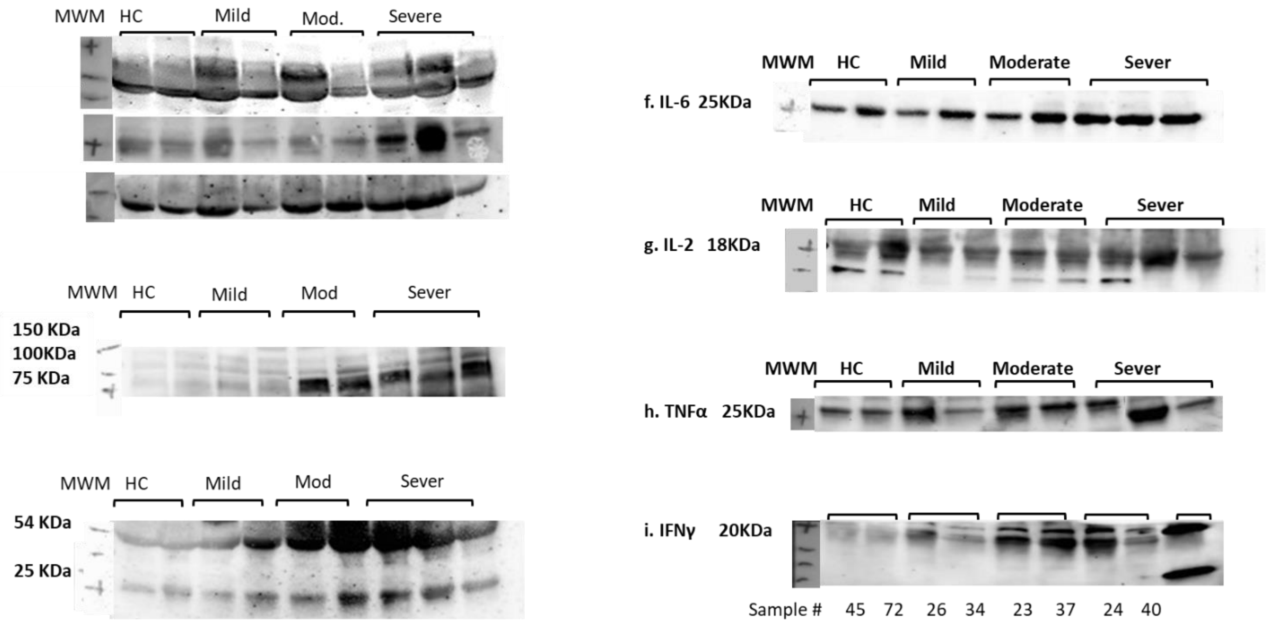

Supplementary figure SM1: Western blot gel images

A representative Western blot image showing the expression of selected proteins in the EV pellet samples of the HCs and the COVID-19 patients. CD81, exosome markers (a), actin protein serves as control (b), ACE and TMPRS SARS-CoV-2 virus entry proteins (c,d). Cytokine content: IL-6 (e), IFNγ (f) TNFα (g), and IL-2 (h).

Supplementary figure SM2: Flow cytometer analysis of patient subgroups and healthy control samples

Megamix Beads size and antibodies	Healthy Control	COVID-19 patients: Mild	Moderate	Severe

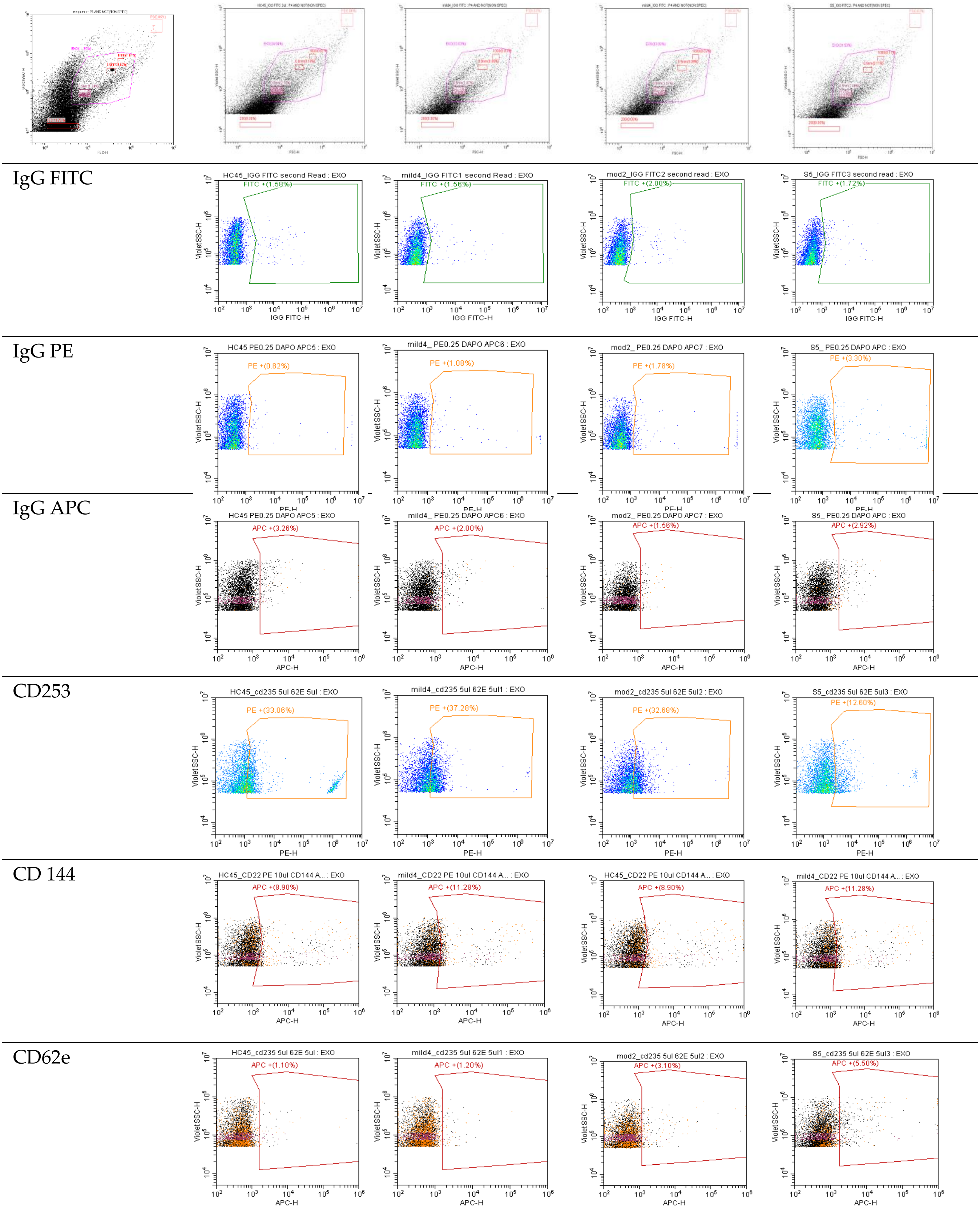

CD41

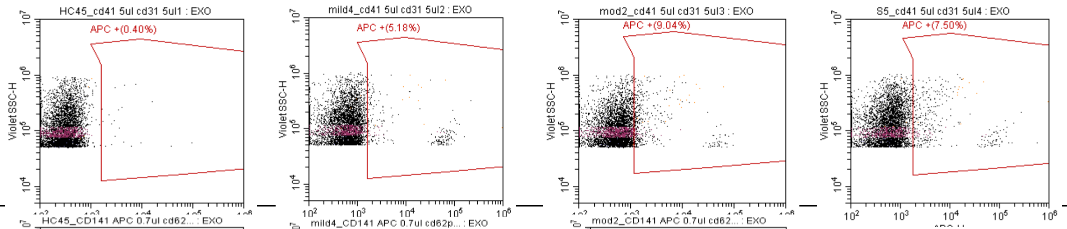

CD62P

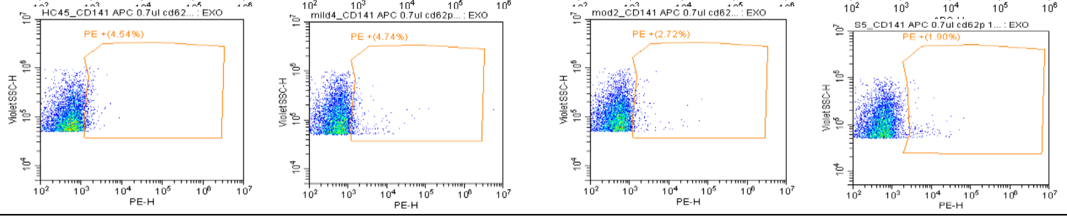

CD31

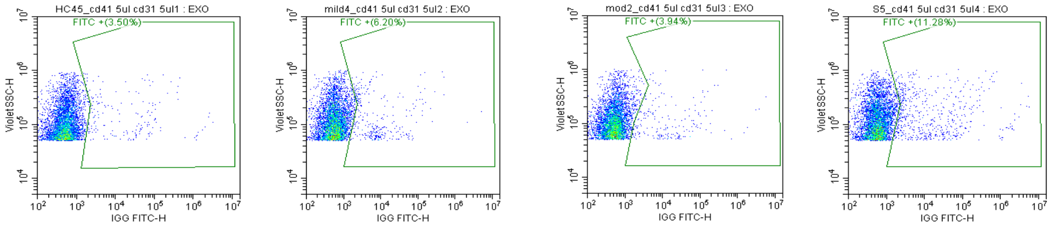

CD142

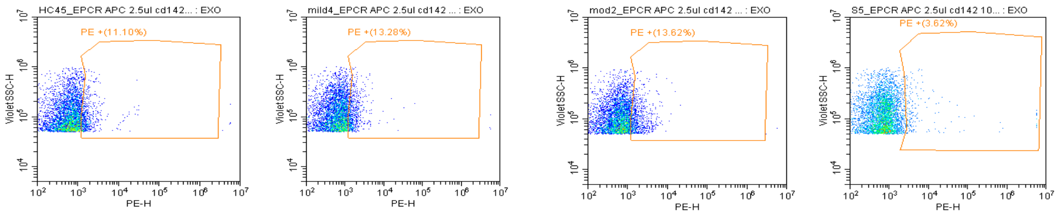

CD141

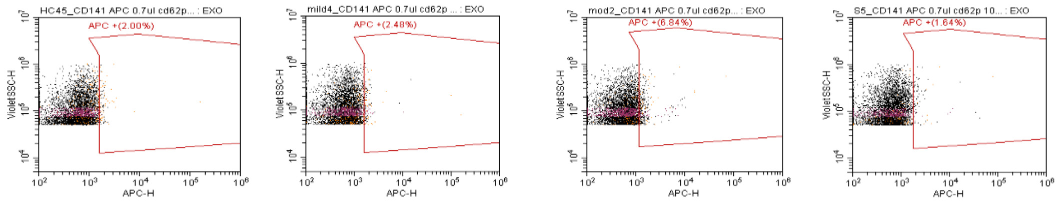

EPCR

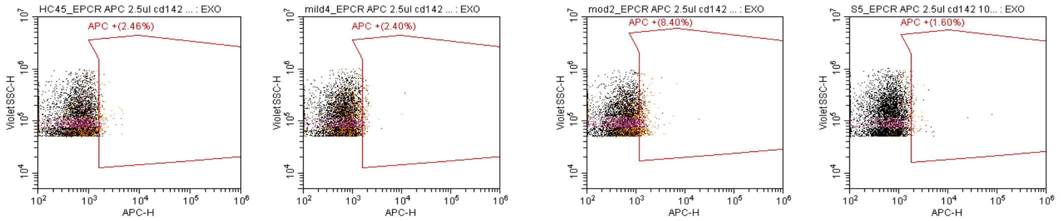

CD4

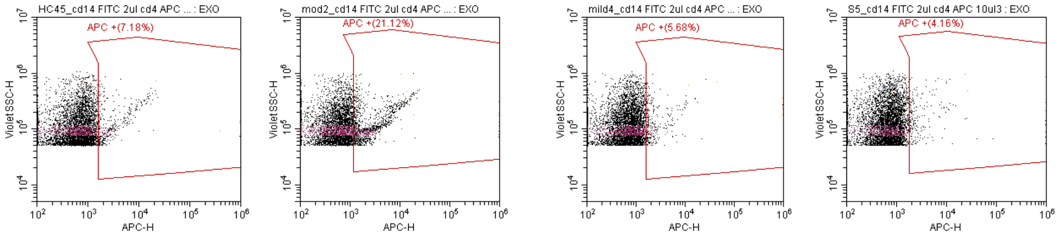

CD8

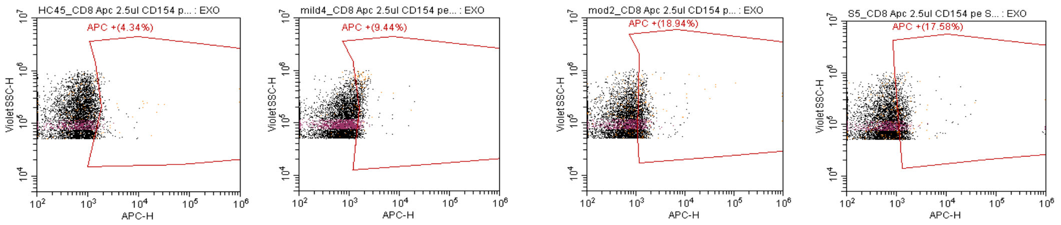

CD28

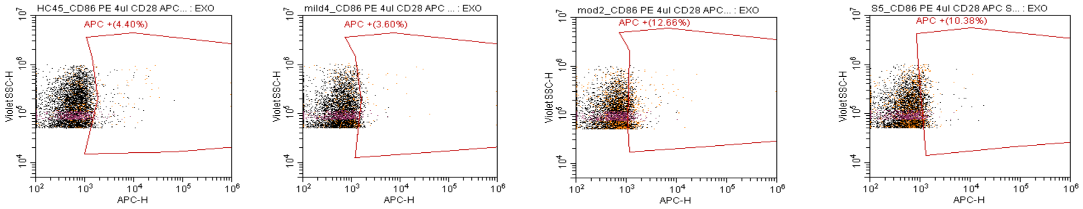

CD154

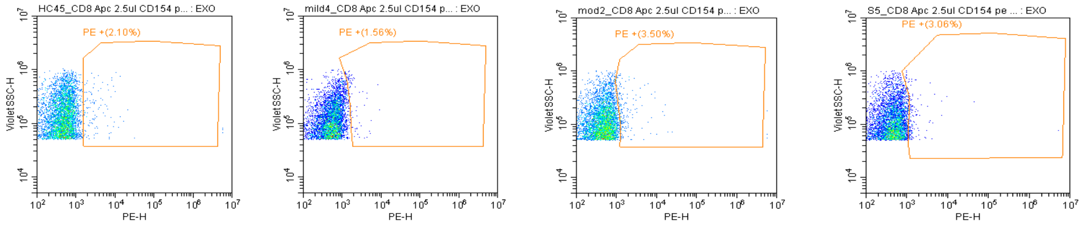

CD14

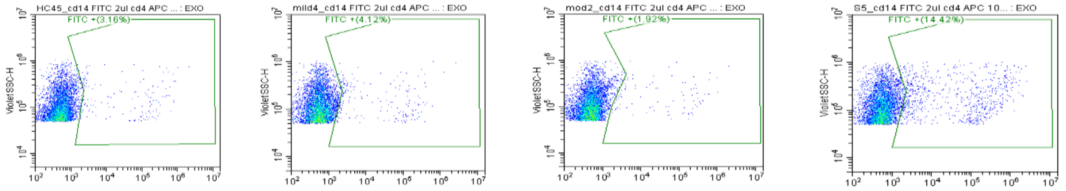

CD22

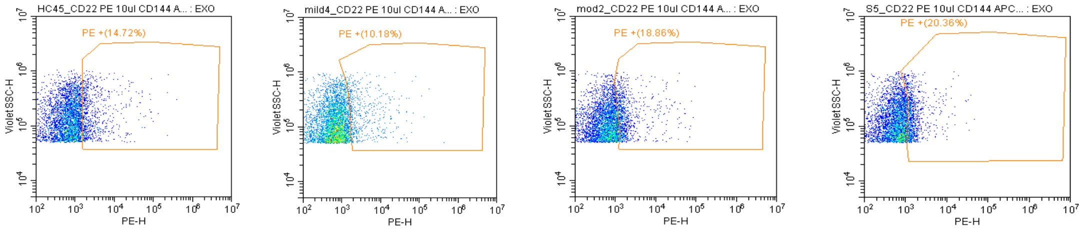

Supplementary figure SM2: Flow cytometer analysis of patient subgroups and healthy control samples

EV gates were set using Megamix, a mix of fluorescent beads (bead size 0.3/0.9/3µm) (a). EV distribution and membrane antigen levels were assessed by flow cytometry using fluorescent antibodies. Representative images of flow cytometer analysis of EVs obtained from the study cohorts, healthy control and COVID-19 patients (mild, moderate, and severe) labeled with isotype controls antibodies (IgG FITC, PE, APC) and fluorescent antibodies against red blood cells (CD253), endothelial markers (CD144, CD62E), platelet and activated platelet marker (CD41, CD62P), coagulation markers: tissue factor (CD142), thrombomodulin (CD141) and endothelial protein C receptor (EPCR), and immune cells markers (CD4, CD8, CD28, CD154, CD14, CD22).
